# Psychometric evaluation of Patient-Reported Outcomes Measurement Information System (PROMIS) in pediatric sickle cell disease in Europe

**DOI:** 10.1007/s00431-026-07113-z

**Published:** 2026-06-03

**Authors:** Maite E. Houwing, Michiel A. J. Luijten, Madieke J. Muntendam, Maud M. van Muilekom, Henriëtte Heijboer, Karin Fijnvandraat, Lotte Haverman, Marjon H. Cnossen

**Affiliations:** 1 Department of Paediatric Haematology, Erasmus MC Sophia Children’s Hospital, University Medical Centre Rotterdam, Wytemaweg 80, 3015 CN Rotterdam, The Netherlands; 2 Emma Children’s Hospital, Child and Adolescent Psychiatry & Psychosocial Care, Amsterdam UMC, University of Amsterdam, Meibergdreef 9, 1105 AZ Amsterdam, The Netherlands; 3 Mental Health and Methodology, Amsterdam Public Health, Meibergdreef 9, 1105 AZ Amsterdam, The Netherlands; 4 Amsterdam Reproduction and Development, Meibergdreef 9, 1105 AZ Amsterdam, The Netherlands; 5 Mental Health and Personalized Medicine, Amsterdam Public Health, Meibergdreef 9, 1105 AZ Amsterdam, The Netherlands; 6 Amsterdam UMC, Emma Children’s Hospital, Pediatric Hematology, University of Amsterdam, Meibergdreef 9, 1105 AZ Amsterdam, The Netherlands; 7 Mental Health and Digital Health, Amsterdam Public Health, Meibergdreef 9, 1105 AZ Amsterdam, The Netherlands; 8 Epidemiology and Data Science, Amsterdam University Medical Center, Vrije Universiteit Amsterdam, Amsterdam, The Netherlands

**Keywords:** Health-related quality of life, Sickle cell disease, Child, Patient-reported outcomes

## Abstract

Sickle cell disease has a profound impact on the physical, mental and social health of affected children. Currently, there is considerable variability among the available patient reported outcomes measures (PROMs) used in children with sickle cell disease, and no consensus has yet been achieved. We aim to assess the psychometric properties of the generic pediatric and proxy Patient-Reported Outcomes Measurement Information System (PROMIS®) measures in children with sickle cell disease living in the Netherlands. Dutch children with sickle cell disease aged 5–17 years old and their caregivers were eligible. The following self-report and proxy-report PROMIS® item banks were evaluated: Anger, Anxiety, Depressive Symptoms, Fatigue, Mobility, Pain Interference, Peer Relationships, Cognitive Functioning, and Global Health. We assessed unidimensionality through confirmatory factory analysis, convergent validity with subscales from the Pediatric Quality of Life Inventory, discriminant validity, reliability, and inter-rater reliability. The study enrolled 102 patients and 102 caregivers, of which 71 were dyads. All item banks displayed sufficient unidimensionality and convergent validity. Discriminant validity was hypothesized and found for “Global Health,” “Mobility” (*d* > 0.3), “Fatigue,” and “Pain Interference” (*d* > 0.3), although some comparisons were non-significant. Reliability was acceptable (*a* > 0.80, SEM < 0.44) for all PROMIS® measures. Inter-rater reliability was moderately-strong for all item bankss (ICC 0.60–0.78) except for “Peer Relationships” (ICC = 0.47, *r* = 0.31) and “Global Health” (ICC = 0.26, *r* = 0.16), which scored lower on correlation.

*Conclusion*: PROMIS® measures displayed sufficient psychometric properties for use in pediatric sickle cell disease care and research. Proxy-reports seem viable as alternative to self-report forms of PROMIS®.

**Table:** 

**What is Known:** • *Sickle cell disease has a profound impact on the physical, mental and social health of affected children and identifying early signs of decline is crucial to intervene before complications arise.* • *No consensus has yet been achieved among the available patient reported outcomes measures (PROMs) used in children with sickle cell disease living in Europe.*
**What is New:** • *This study provides evidence that PROMIS® measures displayed sufficient psychometric properties for use in European pediatric sickle cell disease care and research. * • *Proxy-reports seem viable as alternative to self-report forms of PROMIS®*

**What is Known:**

• *Sickle cell disease has a profound impact on the physical, mental and social health of affected children and identifying early signs of decline is crucial to intervene before complications arise.*

• *No consensus has yet been achieved among the available patient reported outcomes measures (PROMs) used in children with sickle cell disease living in Europe.*

**What is New:**

• *This study provides evidence that PROMIS® measures displayed sufficient psychometric properties for use in European pediatric sickle cell disease care and research. *

• *Proxy-reports seem viable as alternative to self-report forms of PROMIS®*

## Introduction

With millions affected and an estimated 300,000 newborns annually, sickle cell disease (SCD) is the most common monogenetic disease worldwide [1]. In the Netherlands, approximately 2000 individuals have SCD, half of whom are children [2]. Due to painful vaso-occlusive crises and manifestations of organ damage [1, 3], SCD has a substantial impact on daily functioning and well-being of children and their families. Affected children often experience poorer overall health [4, 5], as well as a higher risk of developing depressive symptoms when compared to the general pediatric population [6–9]. Given the heterogeneity in health outcomes in this population, comprehensive care aims to enhance physical, mental and social health by identifying early signs of decline and intervening before complications arise [10]. Monitoring health through patient reported outcome measures (PROMs) covering symptoms, physical, mental and social health [11, 12] is therefore important. Since self- and proxy-reports may diverge, both are needed to fully understand the impact of SCD on a child’s health [12].

Currently, there is considerable variability among the available PROMs used in children with SCD, and no consensus has yet been achieved [13]. Traditional PROMs are often burdensome and outcomes cannot be compared across studies or diseases because a variety of measures are used. Furthermore, many PROMs are available only in English. To provide next generations with standardised generic PROMs with improved reliability and validity, the National Institute of Health (NIH) invested from 2004 onwards in a novel metric system called “Patient Reported Outcomes Measurement Information System” or PROMIS® [14]. Whereas the majority of existing PROMs use classical test theory (CTT) principles, PROMIS® was developed based on the item response theory (IRT), enabling computerized adaptive testing (CAT). CAT dynamically selects the most informative items based on prior responses from extensive item banks. CAT technology, based on IRT results, leads to a measurement that is both concise and precise and can therefore decrease respondent burden [15, 16]. In addition, it makes it possible to compare different measures on a linked, common metric.

PROMIS® does not take a disease-specific approach. There is concern that generic measures may not be appropriate for use by individuals with significant impairment in a specific domain. Its psychometric properties—which refer to validity and reliability of the instrument—need to be established in groups not previously studied [17, 18]. In recent years, the psychometric evaluation of PROMIS® in children with SCD has shown promising results [19–22]. However, most studies have examined a limited number of item banks and focused mainly on the validation of self-report instruments, thereby incorporating only a limited range of proxy measurements [19, 21–23]. Furthermore, all were conducted in North America [19–23], limiting generalizability to European contexts due to differences in healthcare systems and societal structures [24]*.*

Consequently, the aim of this study is to evaluate the psychometric properties of an extensive set of Dutch self- and proxy-report PROMIS® item banks (i.e. Anger, Anxiety, Depressive Symptoms, Fatigue, Mobility, Pain Interference, Peer Relationships, Cognitive Functioning, and Global Health) in children with SCD living in the Netherlands. PROMs should be validated to ensure that they measure the topic (“construct”) that they aim to measure (validity), and that they do this in a reliable way (reliability).

## Methods

### Participants

Patients with SCD aged 8 to 17 years old and caregivers of patients aged 5 to 17 years were considered eligible for study enrolment. Eligible children and their caregivers had to be able to read and speak Dutch or English. Participants received a gift card as reimbursement for their time and efforts. The study was approved by the Medical Ethics Review Committees of the participating centers (W18_265#18.309, W18_265#18.419 and MEC-2018–1508). Prior to enrolment, caregiver written informed consent and child assent were provided.

### Study design

From December 1, 2019, to December 1, 2020, children with SCD and their caregivers were recruited via telephone, email or during outpatient visits at the SCD comprehensive care centres of Erasmus MC Sophia Children’s Hospital and Amsterdam University Medical Centres -Emma Children’s Hospital. Caregivers completed questionnaires on sociodemographic and disease-related characteristics. Both children (self-report) and caregivers (parent proxy-report) completed PROMIS® and the legacy instrument PedsQL 4.0 (Paediatric Quality of Life Inventory 4.0). Measures were completed electronically through the KLIK PROM portal, either at home or in the clinic. Assistance was provided by a trained researcher (M.J.M.) when needed. Caregivers could stay with their child but were instructed not to influence responses. Data including date of birth, SCD genotype, and hospital admissions in the past year, were retrieved from medical records.

### Measures

#### PROMIS®

This study included the following Dutch and English PROMIS® pediatric self-report and proxy-report item banks: Global Health 7 + 2 (v1.0), Cognitive Function (v1.0), Pain Interference (v2.0), Mobility (v2.0), Fatigue (v2.0), Anxiety (v2.0), Anger (v2.0), Depressive Symptoms (v2.0) and Peer Relationships (v2.0). Children aged 8–17 and caregivers of children aged 5–17 completed items referring to the past 7 days, except *Cognitive Function*, which used a 4-week recall period. Items are scored using a 5-point Likert scale ranging from 1 (“never”) to 5 (“almost always”), except for the item bank “Mobility” (ranging from “not able to” to “with no trouble”) and “Global Health” (response categories differ for each item). Scores were calculated using U.S.-calibrated Item Response Theory (IRT) parameters, yielding T-scores (M = 50, SD = 10). Higher scores reflect more of the measured construct, e.g., more fatigue or better cognitive function. In addition, each response pattern receives a standard error of measurement (SEM), indicating the reliability of the measure.

#### PedsQL 4.0

The Pediatric Quality of Life Inventory 4.0 (PedsQL™ 4.0) pediatric self-report and proxy-report, validated in children with SCD [25], were used as legacy instrument. Legacy instruments are PROMs that are widely used and implemented, but have often been developed with classical test theory and therefore do not offer the same benefits as instruments developed with IRT. PedsQL™4.0 is a 23-item multidimensional instrument that measures four core subscales of health including physical, emotional, social and school functioning in children and caregivers. PedsQL™4.0 asks participants to rate how often the subject has experienced a symptom in the past week, scored on a 5-point Likert scale from 1 (“Never a problem”) to 5 (“Almost always a problem”). Higher scores linearly indicate better HRQoL. A total scale score can be computed by averaging all scale scores.

### Psychometric properties

The psychometric properties of PROMIS® self- and proxy-report item banks were analyzed through the assessment of construct validity and reliability. Study sample characteristics were described by descriptive analyses, using the Statistical Package for Social Sciences (SPSS) version 24.0. All other data analyses were performed using R version 3.2.2.

#### Construct validity

Construct validity refers to the extent to which the behavior of the PROM’s scores are consistent with what would be expected from the construct. We assessed construct validity through investigating structural, convergent and discriminant validity.

##### Structural validity

Structural validity measures whether PROM scores adequately reflect the dimensionality of the construct to be measured [26]. As PROMIS® item banks measure a single domain, factorial structure should represent a single, unidimensional factor. Structural validity is usually assessed using confirmatory factor analysis (CFA). In CFA, the more variance of an item can be explained by the hypothesized latent variable (factor), the better the item fits to the construct. A good fit was determined by fit indices of Comparative Fit Index (CFI) and Tucker-Lewis Index (TLI) > 0.95, root mean square error of approximation (RMSEA) < 0.08 and standardized root mean square residual (SRMR) < 0.10. In the event that the unidimensional model did not provide acceptable model fit, modification indices and residual correlations were inspected to identify reasons for model misfit. Unidimensionality was considered sufficient if, in the bi-factor model, the hierarchical omega (ωh) was > 0.80 and the explained common variance (ECV) was > 0.60.

##### Convergent validity

Convergent validity is assessed by means of “hypothesis testing”: determining whether scores of PROMIS® measures correlate with scores on the legacy instrument (i.e., PedsQL™ 4.0) in the way that one would expect. Relationships between PROMIS® and PedsQL™ legacy measures were assessed with Pearson’s correlation coefficient (*r*). A strong correlation (> 0.70 or < −0.70) was expected between PROMIS® T-scores and the sum scores of PedsQL™ scales measuring similar constructs.

##### Discriminant validity

Discriminant validity was assessed by testing whether PROMIS® item banks could distinguish between “known groups” differing in health status*.* Mean scores were compared between children with a severe phenotype (≥ 1 hospital admission in the past year) and those with a less severe phenotype (no admissions), using independent *t*-tests and Cohen’s d to determine effect size. Based on prior research [25–27], significant group differences with at least moderate effect sizes (*d* > 0.3) were expected for Pain Interference, Fatigue, Mobility, and Global Health [27–29].

#### Reliability

Reliability refers to the extent to which the scores on an item set reflect the “true” score on the construct of interest. Scores that are highly reliable are accurate, reproducible, and consistent reflections of the underlying construct that the item set measures. Reliability of the measures was assessed by internal consistency, inter-rater reliability and IRT-based SEM.

##### Internal consistency

Internal consistency reliability measures the extent to which the items in a separate PROMIS® item bank correlate with one another. Internal consistency of the measures was assessed by calculating Cronbach’s coefficient alpha, with a value of 0.8 or greater indicating strong internal consistency.

##### Standard error of measurement

SEM is the standard error of measurement and represents the accuracy (or confidence) we should have in the estimated IRT-based T-score. It is calculated with the formula 1/√I(θ), where I(θ) is total test information at T-score θ). SEM varies across different participants and is therefore dependable on the distribution of scores; however, a mean SEM does provide us with information on the average reliability of the estimates. The mean SEM was calculated for all PROMIS® item banks. A SEM < 4.4, represents a reliability value of ≥ 0.8, which is considered reliable enough for group comparisons.

##### Inter-rater reliability

Inter-rater reliability is the extent to which two or more reporters agree when providing independent ratings [30]. To assess the inter-rater reliability between caregivers and children, intraclass correlations (ICC) were calculated of the paired proxy- and self-reports using a two-way random effect model. The ICC offers an index of absolute agreement given that it takes into account the ratio between subject variability and total variability. An ICC > 0.50 indicates moderate reliability, > 0.70 good reliability and > 0.90 excellent reliability. If the ICC did not display adequate reliability, the raw correlation between the two ratings was also assessed to provide additional confirmation.

## Results

###  Study participants

A total of 102 children with SCD and 102 caregivers consented to participate. Seventy-one patient-proxy dyads completed PROMs. In the potential dyad group, there were some missing assessments from caregivers or children mainly due to non-response. The majority of patients were male (57.9%), with an average age of 11.53 (± 3.27) years. Of the included patients, 21 were aged 5 to 7 years old (all proxy-report), 64 were aged 8 to 12 years old (58 self-report, 48 proxy-report) and 48 were aged 13 to 17 years old (44 self-report, 33 proxy-report). Caregivers were predominately female (72.9%) and biological parents (85.7%). Twelve caregivers did not complete the background information form, resulting in missing data on sociodemographic and disease-related characteristics. Full characteristics per age-group are shown in Table 1.

**Table 1:** Sample characteristics per age-group

	5–7 years	8–12 years	13–17 years	Total sample
	*n* (%)	*n* (%)	*n* (%)	*n* (%)
*n*	21	64	48	133
Self-report only	-	16 (25.0)	15 (31.3)	31 (23.3)
Parent proxy-report only	21 (100)	6 (9.4)	4 (8.3)	31 (23.3)
Self-report and parent proxy-report	-	42 (65.6)	29 (60.4)	71 (53.4)
Missing characteristic	0	5 (7.8)	7 (14.6)	12 (9.0)
*Sociodemographic characteristics patient*				
Age in years, mean (SD)	6.75 (0.758)	10.40 (1.435)	15.13 (1.389)	11.53 (3.271)
Sex, female	14 (66.7)	26 (44.1)	16 (39.0)	56 (42.1)
Special-needs education, yes	1 (4.8)	3 (5.1)	5 (12.2)	9 (6.8)
*Sociodemographic characteristics caregiver*				
Age in years, mean (SD)	33.06 (9.448)	39.94 (8.758)	43.53 (8.618)	39.96 (9.454)
Sex, female	19 (90.5)	47 (79.7)	31 (75.6)	97 (72.9)
Biological parent, yes	20 (95.2)	55 (93.2)	39 (95.1)	114 (85.7)
Number of children, median (min.-max.)	3 (1–6)	3 (1–7)	3 (1–10)	3 (1–10)
Country of birth				
The Netherlands	12 (57.1)	17 (28.8)	14 (34.1)	43 (32.3)
Sub-Saharan Africa	2 (9.5)	15 (25.4)	11 (26.8)	28 (21.1)
Netherlands Antilles or Suriname	7 (33.3)	25 (42.4)	16 (39.0)	48 (36.1)
Other	0	2 (3.4)	0	2 (1.5)
Educational level*				
Low	5 (23.8)	15 (25.4)	15 (36.6)	35 (26.3)
Intermediate	12 (57.1)	30 (50.8)	12 (29.3)	54 (40.6)
High	4 (19.0)	14 (23.7)	14 (34.1)	32 (24.1)
Marital status				
Single	13 (61.9)	29 (49.2)	17 (41.5)	59 (44.4)
Married or in partnership	8 (38.1)	30 (50.8)	24 (58.5)	62 (46.6)
Single-parenting, yes	9 (42.9)	17 (28.8)	16 (39.0)	42 (31.6)
Employment				
Paid employment	14 (66.7)	35 (59.3)	28 (68.3)	77 (57.9)
No paid employment	7 (33.3)	24 (40.7)	13 (31.2)	44 (33.1)
*Disease-related characteristics*				
Haemoglobinopathy				
SS-Sβ0	12 (57.1)	37 (57.8)	35 (72.9)	84 (63.1)
SC-Sβ + -others	9 (42.9)	27 (42.2)	13 (27.1)	49 (36.8)
Crises/year**				
3 or more	4 (19.4)	17 (28.8)	16 (39.0)	37 (27.8)
mean (SD)	1.19 (1.632)	1.66 (2.397)	3.02 (3.883)	2.04 (2.962)
Admissions/year**				
1 or more	1 (4.8)	19 (32.2)	20 (48.9)	40 (30.1)
mean (SD)	0.10 (0.436)	0.56 (1.038)	1.22 (2.242)	0.70 (1.547)
Physical comorbidity, yes	0	8 (13.6)	4 (9.8)	12 (9.0)
Psychological comorbidity, yes	2 (9.5)	6 (10.2)	5 (12.3)	13 (9.8)

^*^Highest educational level completed. Low: Primary education, lower vocational education, lower or middle general secondary education; Intermediate: middle vocational education, higher secondary education, pre-university education; High: higher vocational education, university

^**^Number of crises and admissions in the year prior to time-point of measurement

### Construct validity

#### Structural validity

The unidimensionality of most PROMIS® measures was sufficient (CFI/TLI > 0.95, RMSEA < 0.08, SRMR < 0.10) except for the self- and proxy-report “Mobility” item bank and the proxy-report “Cognitive Function” item bank, which displayed a high SRMR and low hierarchical omega and ECV values (Table 2).

**Table 2:** The unidimensionality of the PROMIS*® measures*

	Fit Indices					
	CFI	RMSEA	TLI	SRMR	Omega H	ECV
*PROMIS® item banks self-report*						
Anger	0.969	0.165	0.958	0.076	0.827	0.711
Anxiety	0.984	0.071	0.981	0.072	0.841	0.765
Depressive Symptoms	0.973	0.099	0.968	0.076	0.717	0.618
Fatigue	0.963	0.082	0.960	0.086	0.811	0.703
Mobility	0.973	0.048	0.971	0.542	0.495	0.440
Pain Interference	0.979	0.086	0.976	0.064	0.864	0.791
Peer Relationships	0.974	0.094	0.970	0.063	0.794	0.741
Perceived Cognitive Function	0.951	0.061	0.949	0.102	0.875	0.750
Global Health	0.986	0.139	0.979	0.084	0.723	0.604
*PROMIS® item banks parent proxy-report*						
Anger	0.994	0.125	0.987	0.038	0.815	0.779
Anxiety	0.980	0.103	0.976	0.074	0.737	0.632
Depressive Symptoms	0.991	0.078	0.989	0.062	0.817	0.717
Fatigue	0.988	0.076	0.986	0.056	0.872	0.802
Mobility	0.991	0.042	0.990	0.450	0.651	0.507
Pain Interference	0.991	0.147	0.989	0.040	0.903	0.984
Peer Relationships	0.978	0.104	0.974	0.060	0.822	0.753
Perceived Cognitive Function	0.927	0.085	0.924	0.131	0.521	0.422
Global Health	0.986	0.139	0.979	0.084	0.723	0.604

*CFI* comparaitive fit index, values > 0.95 are deemed sufficient, *TLI* Tucker-Lewis index, values > 0.95 are deemed sufficient, *RMSEA* root mean squared error of approximation, values < 0.10 are deemed acceptable, *Omega H* omega hierarhical, values > 0.8 are deemed sufficient, *ECV* explained common variance, values > 0.6 are deemed sufficient

#### Convergent validity

Table 3 shows the correlations between PROMIS® and the legacy instrument, PedsQL™ 4.0. All PROMIS® measures were significantly correlated with related subscales of PedsQL™ 4.0, except for the self-report “Global Health” item bank which did not correlate with any of the subscales of PedsQL™4.0 (including the hypothesized total score). The correlation of the “Fatigue” item bank with PedsQL™4.0 “Physical Functioning” subscale was lower than expected for both self- and proxy-report (− 0.441 and − 0.399 respectively). Furthermore, the Peer Relationships item bank demonstrated only a weak correlation (*r* = 0.283) with the “Social Functioning” subscale of PedsQL™4.0. Correlations were similar between self- and proxy-report.

**Table 3:** Convergent validity of the PROMIS® item banks with the PedsQL 4.0 (sub)scales

	*PedsQL (sub)scales*									
	Physical functioning		Emotional functioning		Social functioning		School functioning		Total	
	Pearson r	*p*-value	Pearson r	*p-*value	Pearson r	*p*-value	Pearson r	*p*-value	Pearson r	*p*-value
*PROMIS® item banks* *self-report*										
Anger	− 0.056	0.578	** − 0.546**	< 0.001	− 0.272	0.006	− 0.245	0.013	− 0.310	0.002
Anxiety	− 0.456	< 0.001	** − 0.642**	< 0.001	− 0.554	< 0.001	− 0.326	< 0.001	− 0.605	< 0.001
Depressive Symptoms	− 0.235	0.017	** − 0.566**	< 0.001	− 0.341	< 0.001	− 0.288	0.003	− 0.426	< 0.001
Fatigue	** − 0.441**	< 0.001	− 0.478	< 0.001	− 0.407	< 0.001	− 0.389	< 0.001	− 0.533	< 0.001
Mobility	**0.745**	< 0.001	0.301	0.002	0.502	< 0.001	0.357	< 0.001	0.631	< 0.001
Pain Interference	** − 0.505**	< 0.001	− 0.477	< 0.001	− 0.448	< 0.001	− 0.437	< 0.001	− 0.582	< 0.001
Peer Relationships	0.208	0.036	0.258	0.009	**0.283**	0.004	0.238	0.016	0.295	0.003
Cognitive Function	0.540	< 0.001	0.464	< 0.001	0.588	< 0.001	**0.701**	< 0.001	0.699	< 0.001
Global Health	0.14	0.162	− 0.058	0.561	0.027	0.784	0.124	0.215	**0.081**	0.416
*Parent proxy-report*										
Anger	− 0.077	0.440	** − 0.437**	< 0.001	− 0.272	0.006	− 0.232	0.019	− 0.299	0.002
Anxiety	− 0.281	0.004	** − 0.602**	< 0.001	− 0.472	< 0.001	− 0.331	< 0.001	− 0.525	< 0.001
Depressive Symptoms	− 0.243	0.014	** − 0.574**	< 0.001	− 0.476	< 0.001	− 0.346	< 0.001	− 0.505	< 0.001
Fatigue	** − 0.399**	< 0.001	− 0.222	0.025	− 0.376	< 0.001	− 0.418	< 0.001	0.474	< 0.001
Mobility	**0.508**	< 0.001	0.257	0.009	0.420	< 0.001	0.383	< 0.001	0.537	< 0.001
Pain Interference	** − 0.261**	0.008	− 0.265	0.007	− 0.306	0.002	− 0.165	0.097	− 0.329	0.001
Peer Relationships	0.076	0.445	0.370	< 0.001	**0.283**	< 0.004	0.190	0.056	0.272	0.006
Cognitive Function	0.101	0.369	0.407	< 0.001	0.117	0.296	**0.535**	< 0.001	0.345	0.002
Global Health	0.319	0.001	0.281	0.004	0.176	0.076	0.418	< 0.001	**0.395**	< 0.001

Bold correlations were hypothesized to be associated, highly correlated (sub)scales

#### Discriminant validity

Table 4 shows that both PROMIS® self- and proxy-report “Fatigue,” “Mobility,” “Pain Interference,” and “Global Health” item banks were able to distinguish between phenotype severity as hypothesized, based on the effect size cut-off of *d* > 0.3. Other self-report item banks did not show significant mean differences between severity groups, except for a moderate effect size for “Depressive Symptoms.” Contrarily, for the proxy-report moderate effect sizes were also found for “Anger,” “Depressive Symptoms,” “Anxiety,” and “Peer Relationships.”

**Table 4:** Discriminant validity of the PROMIS® item banks between patients with a severe phenotype (≥ 1 hospital admission) and less severe phenotype

	Severe phenotype	Less severe phenotype			
	Mean	Mean	Mean difference (SE)	*p*-value	Cohen’s D (95% CI)
*PROMIS® item banks self-report, n*	36	54			
Anger	45.09	43.90	1.18 (2.76)	0.670	0.09 (− 0.33;0.52)
Anxiety	46.04	42.69	3.35 (2.49)	0.181	0.29 (− 0.14;0.72)
Depressive Symptoms	45.49	41.95	3.54 (2.37)	0.140	0.32 (− 0.11;0.75)
** Fatigue**	**46.66**	**41.80**	**4.87 (2.74)**	**0.079**	**0.38 (− 0.05;0.81)**
** Mobility**	**47.58**	**53.39**	** − 5.80 (1.87)**	**0.003**	** − 0.67 (− 1.10; − 0.24)**
** Pain Interference**	**52.73**	**47.35**	**5.38 (2.88)**	**0.066**	**0.40 (− 0.03;0.83)**
Peer Relationships	48.79	51.29	− 2.50 (2.29)	0.278	− 0.23 (− 0.66;0.19)
Cognitive Function	48.86	50.14	1.28 (1.55)	0.414	− 0.18 (− 0.60;0.25)
** Global Health**	**41.15**	**45.38**	** − 4,21 (1.75)**	**0.018**	** − 0.49 (− 0.92; − 0.06)**
*PROMIS® item banks parent proxy-report, n*	32	70			
Anger	44.42	40.07	− 4.35 (2.61)	0.099	0.36 (− 0.07;0.78)
Anxiety	47.74	42.61	− 5.13 (2.18)	0.021	0.50 (0.08;0.93)
Depressive Symptoms	47.11	38.51	− 8.59 (2.39)	0.001	0.83 (0.40;1.25)
** Fatigue**	**50.15**	**38.88**	** − 11,27 (2.36)**	** < 0.001**	**1.02 (0.60;1.44)**
** Mobility**	**46.43**	**53.41**	**6.98 (1.92)**	** < 0.001**	** − 0.77 (− 1.20; − 0.35)**
** Pain Interference**	**54.59**	**48.51**	** − 6.07 (2.66)**	**0.025**	**0.49 (0.06;0.91)**
Peer Relationships	47.10	50.08	2.98 (2.01)	0.141	− 0.32 (− 0.74;0.11)
Cognitive Function*	50.25	50.52	0.27 (1.55)	0.863	− 0.04 (− 0.49; − 0.42)
** Global Health**	**36.49**	**43.34**	**6.86 (1.88)**	** < 0.001**	** − 0.78 (− 1.20; − 0.35)**

Bold means were hypothesized to differ significantly between severity groups

^*^PROMIS**®** Perceived cognitive function: severe phenotype *n* = 31, less severe phenotype *n* = 50

### Reliability

####  Internal consistency

All PROMIS® item banks demonstrated acceptable levels of internal consistency with alpha reliability coefficients above 0.80 (Table 5).

**Table 5:** Internal consistency and standard error of measurement of PROMIS® item banks

	Cronbach’s alpha	Average SEM
*PROMIS® self-report item banks*		
Anger	0.95	4.19
Anxiety	0.95	4.23
Depressive Symptoms	0.95	4.24
Fatigue	0.96	3.24
Mobility	0.94	4.24
Pain Interference	0.97	3.04
Peer Relationships	0.95	3.50
Cognitive Function	0.97	1.26
Global Health	0.88	3.16
*PROMIS® parent proxy-report item banks*		
Anger	0.93	4.40
Anxiety	0.95	3.92
Depressive Symptoms	0.96	4.19
Fatigue	0.98	3.19
Mobility	0.96	4.39
Pain Interference	0.85	3.27
Peer Relationships	0.95	2.82
Cognitive Function	0.97	1.40
Global Health	0.88	3.08

#### Standard error of measurement

All PROMIS® item banks showed acceptable SEMs (< 4.4) to allow for group comparisons; see Table 5.

#### Inter-rater reliability

Table 6 shows ICC between PROMIS® self- and proxy-report item banks for the entire sample. Except for the “Global Health” and “Peer Relationships” item bank, PROMIS® self- and proxy-report measures displayed at least moderate inter-rater reliability (range ICC = 0.595–0.784). “Global Health” displayed poor agreement (ICC = 0.264). Investigating the correlation between the two ratings confirmed this (*r* = 0.158). “Peer Relationships” displayed borderline moderate reliability (ICC = 0.466) with a correlation between ratings of *r* = 0.306, indicating a weak correlation.

**Table 6:** Inter-rater reliability between self- and parent proxy-report of PROMIS® item banks

	ICC
*PROMIS® item banks*	
Anger	0.595 (0.354–0.747)
Anxiety	0.656 (0.449–0.785)
Depressive Symptoms	0.742 (0.587–0.839)
Fatigue	0.689 (0.500–0.806)
Mobility	0.613 (0.379–0.759)
Pain Interference	0.608 (0.371–0.755)
Peer Relationships	0.466 (0.146–0.666)
Cognitive Function	0.784 (0.654–0.866)
Global Health	0.264 (− 0.157–0.535)

## Discussion

We aimed to investigate whether pediatric PROMIS® self- and proxy-report measures demonstrate adequate psychometric properties when applied in a Dutch population of children with SCD. Findings extend evidence obtained in North America for the validity and reliability of PROMIS®. Most item banks displayed sufficient unidimensionality and correlated strongly with the related legacy instrument (PedsQL 4.0) and demonstrated adequate discriminant validity—particularly for the item banks “Fatigue,” “Pain Interference,” “Mobility,” and “Global Health.” All item banks provided reliable, accurate measurements for both self- and proxy-report. The inter-rater reliability was moderately strong between parents and children, except for peer relations and global health.

In general, findings align with prior studies in chronically ill children. For example studies in children with juvenile idiopathic arthritis showed similar results regarding unidimensionality, reliability [31] and inter-rater reliability [32]. Nevertheless, for “Mobility” and “Cognitive Functioning,” skewness and ceiling effects were observed—consistent with earlier PROMIS® validation studies in children with SCD [20]. Patients and their caregivers completed measures in the outpatient clinic or at home where minimal impairment might be expected (in contrast to children who are hospitalized). However, it may also be due to the fact that these patients simply did not have any impairments in these domains or that the measures were not able to capture differences that are milder in nature. This aligns with the described sociodemographic characteristics of the sample, as amount of hospitalization was low. This may have influenced the unidimensionality of these item banks.

Overall, convergent validity was deemed sufficient and comparable to previous studies validating PROMIS® in the general pediatric population [33, 34]. Compared to these studies, correlations between instruments that assess the same domain were similar (*r* > 0.50), In addition, we found weaker correlation between Peer Relationships and the PedsQL Social Functioning scale This can be explained as these domains are related, but separate subdomains of social health. This has previously been mentioned in the validation paper in the Dutch general population [34] and during the initial development of the item bank [35]. During (sub)domain elicitation, researchers were unable to develop a unidimensional item bank without separating relationships with peers from social functioning with adults [35].

However, some unexpected findings emerged. PROMIS® “Fatigue” and PedsQL™4.0 “Physical Functioning” did not correlate as highly as expected and “Fatigue” item bank correlated more strongly with the total PedsQL™4.0 score. This could be explained by the fact that “Physical Functioning” of PedsQL™4.0 does not necessarily assess fatigue (only one item focuses on fatigue). Therefore it may have been better to include PedsQL™4.0 Multidimensional Fatigue Scale [36]. Lastly, PROMIS® self-report “Global Health” did not correlate with PedsQL™4.0 total score, likely due to differences in the way items are formulated: PROMIS® emphasizes positive health, while PedsQL™ 4.0 focuses on impairments. Children are asked to rate their own health, which may be influenced by subjective feelings of self-criticism or social comparisons [33]. More explicitly, children with a chronic disease may have adjusted to their disease and it may therefore be more difficult for them to rate their own health, as we see higher correlations between PedsQL™4.0 self-report total score and PROMIS® “Global Health” item bank scores as reported by caregivers.

Caregivers of children with severe disease phenotype had significantly higher scores on almost all PROMIS® proxy-report item banks when compared to caregivers of children with less severe phenotype. However, in children, this effect was only evident for “Fatigue,” “Mobility,” “Pain Interference,” and “Global Health” self-report item banks. These discrepancies likely reflect underreporting by children or overreporting by caregivers, which is especially common for emotional and social problems as shown in previous literature [37]. Nonetheless, the inter-rater reliability of PROMIS® item banks was good and comparable to ICC reported in a previous study of dyads in juvenile idiopathic arthritis [31, 34]. However, in both studies the measured ICC may have been influenced by the inclusion of mainly young children and fewer adolescents, as strong ICC between child-caregiver dyads are quite uncommon. Especially with regards to mental health, imperfect agreement between self-report and proxy-report has been consistently documented [38]. With younger children, however, caregivers might be more aware of the possible negative consequences of the illness. In addition, our data collection partly took place during COVID-19 pandemic when children were likely spending more time at home and therefore easier to observe by parents. A third possible explanation could be that children did not complete the self-report measures themselves, but instead caregivers completed all PROMs. However, this is unlikely, as PROMIS® “Global Health” item bank did not have a high ICC. As previously mentioned, “Global Health” item bank may be more subjective due to the way the questions are asked, which likely impacted the results of the inter-rater reliability for this measure.

Although our study had a robust study design, it has some limitations. Firstly, we have a double-site convenience sample, which might limit generalizability. However, the sample is moderate in size and includes quite a large number of patient-proxy dyads and has a similar distribution to other studies in terms of socioeconomic status, sex and disease phenotype. Secondly, this study is limited to the Dutch population. Therefore, we cannot assume that the measures would have the same test characteristics in other populations. Thirdly, although cross-cultural validity was assessed previously and found to be sufficient for PROMIS® self-report in a large sample of Dutch children representative for the Dutch population [34], cross-cultural validity for proxy-reports has not been formally established. Unfortunately, we could not perform these analyses due to small participant numbers. Lastly, this study was partly conducted during the COVID-19 pandemic. We are, therefore, not able to differentiate between the effect of SCD and possible COVID-19 effects. This could have influenced, for example, the measured inter-rater reliability.

In conclusion, this study provides evidence for sufficient psychometric properties of pediatric and caregiver-proxy PROMIS® item banks. PROMIS® measures are valid and reliable indicators of patient reported outcomes among children aged 5–17 with SCD living in Europe. When administered as CAT, PROMIS® can provide brief assessments of patient reported outcomes important to children with SCD and their caregivers. Further clinical validation studies, especially regarding responsiveness of the item banks to changes in treatment, are warranted before use in clinical practice.

## Data Availability

No datasets were generated or analysed during the current study.

## Abbreviations

SCD: Sickle cell disease
HRQoL: Health-related quality of life
PROMIS®: Patient-Reported Outcomes Measurement Information System®
PROMs: Patient-reported outcomes measures
NIH: National Institute of Health
CTT: Classical test theory
IRT: Item response theory
CAT: Computer adaptive testing
PedsQL 4.0: Pediatric Quality of Life Inventory 4.0
SPSS: Statistical Package for Social Sciences
CFA: Confirmatory factor analysis (statistics)
CFI: Comparative Fit Index (statistics)
TLI: Tucker-Lewis Index (statistics)
RMSEA: Root mean square error of approximation (statistics)
SRMR: Standardized root mean square residual (statistics)
ECV: Explained common variance (statistics)
SEM: Standard error of measurement (statistics)
ICC: Intraclass correlations (statistics)
